# Retrosigmoid approach assisted by high-resolution computed tomography: a cost-effective technique to identify the transverse and sigmoid sinus transition

**DOI:** 10.1186/s41016-020-00192-3

**Published:** 2020-07-01

**Authors:** Runfeng Wang, Zhiguo Zhang, Zhihong Li, Yan Qu

**Affiliations:** Tangdu Hospital, Air Force Military Medical University, No. 1, Xisi Road, Baqiao District, Xi’an City, China

**Keywords:** Retrosigmoid approach, Transverse and sigmoid sinus transition, Lambdoid suture, Occipitomastoid suture, LZI, Mastoid emissary foramen

## Abstract

**Background:**

When utilizing the retrosigmoid approach (RA), accurately identifying the transverse and sigmoid sinus transition (TSST) is a key procedure for neurosurgeons, especially in developing countries restricted by the lack of expensive devices, such as the neural navigation system and the three-dimensional volumetric image-rendered system. Before operations, a computed tomography scan is a common and cost-effective method of checking patients who suffer lesions located at the cerebellopontine angle. Therefore, we present a technique using only high-resolution computed tomography to identify the transverse and sigmoid sinus transition.

**Methods:**

This retrospective study included 35 patients who underwent retrosigmoid approach operations to resect an acoustic neurinoma with the assistance of our technique. In brief, our technique contains 4 steps: (1) All patients’ 1-mm, consecutive, high-resolution computed tomographic images that clearly displayed landmarks, such as the inion, lambdoid suture, occipitomastoid suture, and the mastoid emissary foramen, were investigated initially. (2) We selected two particular slices (A and B) among all of these high-resolution computed tomographic images in which scanning planes were parallel with the line drawn from the root of the zygoma to the inion (LZI). Slice A contained both the root of the zygoma and the inion simultaneously, and slice B displayed the mastoid emissary foramen. (3) Four points (*α*, *β*, *γ*, *δ*) were arranged on slices A and B, and point *α* was located at the inner surface of the skull, which represents the posterior part of the sulci of the sigmoid sinus. Point *β* was located at the outer surface of the skull, and the line connecting them was perpendicular to the bone. Similarly, on slice B, we labeled point *γ* as the point that represents the posterior part of the sulci of the sigmoid sinus at the inner surface and point *δ* as the point located at the outer surface of the skull, and the line connecting them was also perpendicular to the bone. The distances between point *β* and the lambdoid suture/occipitomastoid suture and between point *δ* and the mastoid emissary foramen were calculated for slices A and B, respectively. (4) During the operation, a line indicating the LZI was drawn on the bone with ink when the superficial soft tissue was pushed away, and this line would cross the lambdoid suture/occipitomastoid suture. With both the crosspoint and the distance obtained from the high-resolution CT images, we could locate point *β*. We also used the same method to locate point *δ* after revealing the mastoid emissary foramen. The line connecting point *β* and point *δ* indicated the posterior border of the sigmoid sinus, and the intersection between the line and LZI indicated the inferior knee of the transverse and sigmoid sinus transition (TSST).

**Results:**

All 35 patients underwent the RA craniectomies that were safely assisted by our technique, and neither the sigmoid sinus nor the transverse sinus was lacerated during the operations.

**Conclusion:**

Our cost-effective technique is reliable and convenient for identifying the transverse and sigmoid sinus transition (TSST) which could be widely performed to guarantee the safety of RA craniectomy.

## Background

Retrosigmoid approach is a routine and effective method for neurosurgeons to resect lesions located at the cerebellopontine angle. In contrast to the transverse sinus, the sigmoid sinus is embedded into a bone groove, which could be easily lacerated during suboccipital craniectomy. Therefore, accurately identifying the border of the sigmoid sinus is an essential procedure during operation. For developed countries, with the assistance of the neural navigation system or the three-dimensional volumetric image-rendered system [1-4], neurosurgeons can precisely and conveniently identify the transverse and sigmoid sinus transition (TSST). However, for the majority of the countries worldwide, when these high-tech and expensive devices are absent, safely performing the standard RA remains a challenge. A computed tomography scan is a common and irreplaceable preoperative method of checking patients who suffer from acoustic neurinoma, and a series of anatomical studies have revealed the relationships between the surface landmarks and the underlying structures regarding RA [5-8]. Therefore, we hypothesized that this cost-effective and reliable technique combined with the existing anatomical knowledge and high-resolution CT images identify the location of the TSST.

## Methods

**Fig. 1:**
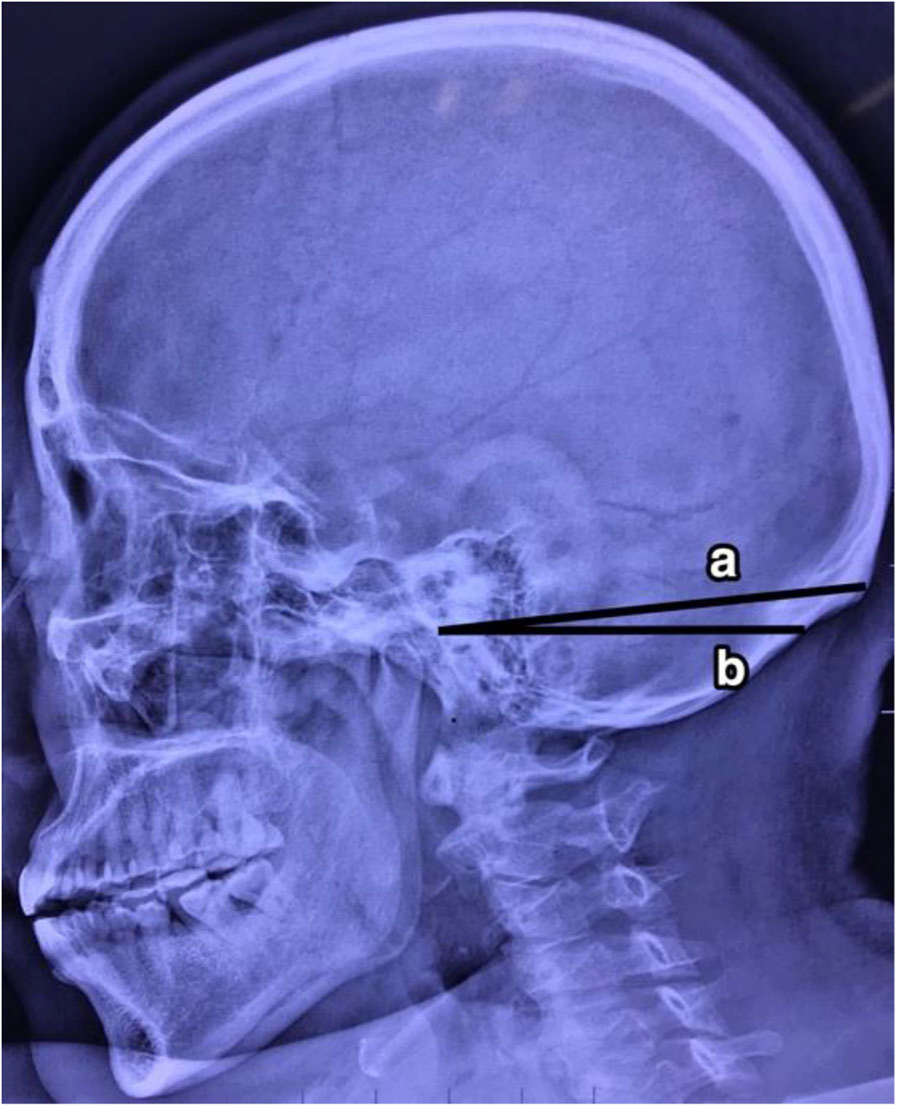
Scanning plane. This figure shows our scanning plane (**a**) that is parallel with the line drawn from the root of the zygoma to the inion, which is at an angle from the usual scanning plane (**b**)

**Fig. 2:**
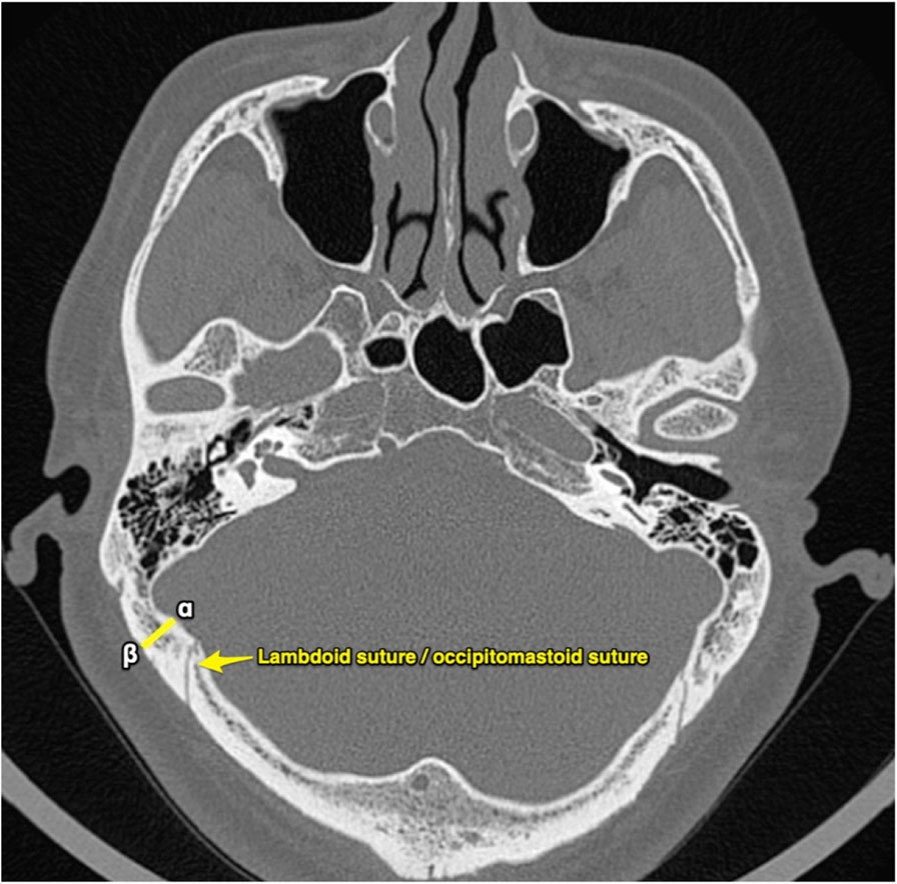
Slice A. This figure shows slice A, which displays the root of the zygoma and the inion simultaneously, the locations of point α and point β are displayed, and the line that connects them is perpendicular to the skull. The distance between point β and the lambdoid suture/occipitomastoid suture on the outer surface of the skull should be measured

**Fig. 3:**
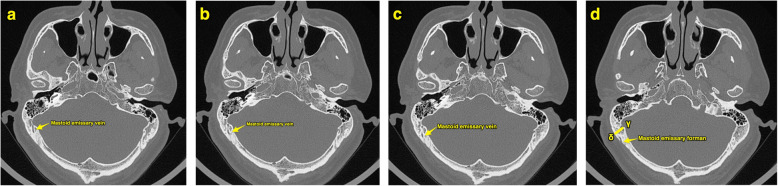
The course of mastoid emissary vein and slice B. The course of mastoid emissary vein can be traced through the continuous CT images (**a**–**c**). The slice B (**d**) should reveal the mastoid emissary foramen and the locations of point γ and point δ. The distance between point δ and the mastoid emissary foramen should be measured

This study was approved by the Medical Ethics Committee of the Second Affiliated Hospital of the China Air Force Military Medical University. From January to December 2018, 35 patients (22 males, 13 females; mean age 51 years [range 26–71 years]) with an acoustic neurinoma underwent operations performed through RA were enrolled in this study. Among these patients, 16 pathologies were located on the left side, and the other pathologies were located on the right side. All patients underwent a preoperative high-resolution CT scan (General Electric Healthcare, Chalfont St. Giles, England). Usually, the scan is performed in parallel with the Frankfurt horizontal plane (FHP). For the purpose of our study, we adjusted the angle of the scan to be parallel with the line drawn from the root of the zygoma to the inion (Fig. 1). After the angle was checked, a series of consecutive images with a thickness of 1 mm were obtained. When these images were investigated, 2 particular slices labeled slice A and slice B were selected. On slice A, both the root of the zygoma and the inion were displayed simultaneously, and on slice B, the mastoid emissary foramen was revealed. Then, we distributed 4 points (*α*, *β*, *γ*, *δ*) on slices A and B. Regarding slice A, point *α* was located at the inner surface of the skull, which represents the posterior border of the sulci of the sigmoid sinus. The line drawn from point *α*, which was perpendicular to the bone, crossed the outer surface of the skull. The point at which they crossed was labeled point *β*. Similarly, on slice B, point *γ* and point *δ* were arranged at the inner surface and outer surface of the skull, respectively (Figs. 2 and 3). There was an angle between the scanning plane and the path of the lambdoid suture and the occipitomastoid suture; thus, both of the sutures were vividly revealed on the high-resolution axial CT images. Subsequently, the distance between point *β* and the lambdoid suture/occipitomastoid suture and between point *δ* and the mastoid emissary foramen on the outer surface of the skull could be measured on slices A and B, respectively.

All surgeries were performed with the patient in the lateral prone position and the mastoid at the highest point, and a Mayfield clamp system was used to stabilize the head. When these skull landmarks, such as the mastoid emissary foramen, lambdoid suture, occipitomastoid suture, and the mastoid, were exposed, an indelible ink line was drawn on the bone representing the LZI, which crossed the lambdoid suture/occipitomastoid suture. Marked this crosspoint and combined both the location of this crosspoint and the data acquired preoperatively on slice A, point *β* could be precisely determined by the utilization of gauge. Similarly, point *δ* could be determined with the assistance of the location of the mastoid emissary foramen and the preoperative data acquired on slice B. Point *β* and *δ* should be marked by the indelible ink. We hypothesized that the lateral part of the line that connected the inion and the root of the zygoma (LZI) represents the most distal part of the transverse sinus and the line that connected points *β* and *δ* represents the posterior border of the sigmoid sinus. Therefore, the intersection between these two lines would indicate the inferior knee of the transverse and sigmoid sinus transition (TSST). During the operation, the first drill hole was positioned on the intersection. Subsequently, we performed a standard RA craniectomy and recorded the location of the transverse sinus, the posterior border of the sigmoid sinus, and the TSST. A grid frame with subdivisions with a dimension of 1 × 1 cm^2^ was utilized to evaluate the accuracy of our technique centered over our projected intersection point, and the horizontal and perpendicular lines of the grid that were parallel with the transverse sinus and the sigmoid sinus, respectively, and the actual locations of the TSST were recorded. The data were expressed as a percentage of the actual TSST located in each subdivision on the grid.

## Results

**Fig. 4:**
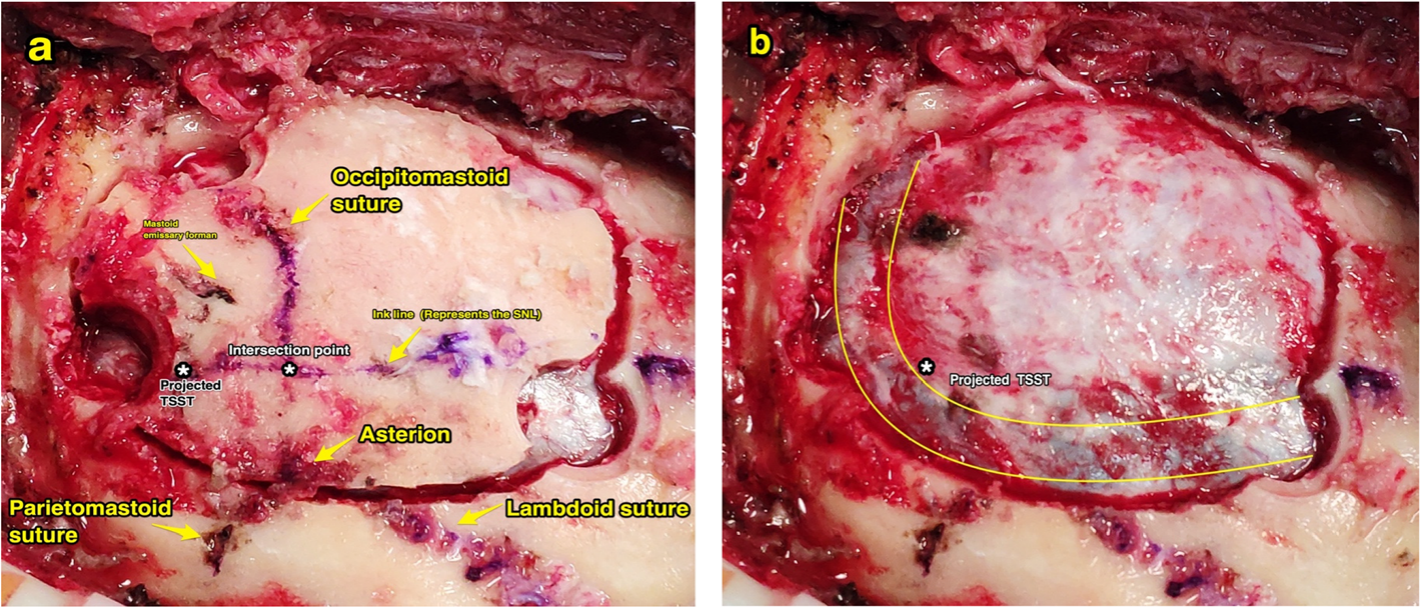
**a** Actual views during the operation (right side), which reveal the relationship between the projected TSST and the actual TSST and the intersection point at which the SNL meets the lambdoid suture/occipitomastoid suture, and the landmarks of the skull have also been displayed. The ink line drawn on the surface of the skull represents the SNL. To obviously skeletonize the transverse sinus and the sigmoid sinus, the first drill hole was positioned slightly lateral to the projected TSST. **b** The actual views during operation when craniectomy was completed

All 35 patients underwent the RA craniectomies that were safely assisted by our technique, and neither the sigmoid sinus nor the transverse sinus was lacerated during the operations. There were 2 cases of emissary vein bleeding during the craniectomies that were coagulated efficiently. Figure 4 displays the actual views during operations. The distance between point *β* and the lambdoid/occipitomastoid suture on the outer surface of the skull which were measured by the gauge was 1.67 ± 0.36 cm on the left and 1.88 ± 0.23 cm on the right, and the distance between point *δ* and the mastoid emissary foramen was 0.63 ± 0.38 cm on the left and 0.78 ± 0.26 cm on the right. Regarding these data, there were no significant differences between the left and right sides (*P* < 0.05).

There were 2 (2/6) projected transverse sinuses on the left and 3 (3/6) on the right that were inferior by more than 0.5 cm to the actual transverse sinuses. Only 1 (1/6) projected transverse sinus was superior by more than 0.5 cm to the actual transverse sinus, which was located on the right.

Only 1 (1/35) projected sigmoid sinus was posterior by more than 0.5 cm to the actual sigmoid sinus, which was located on the right.

**Fig. 5:**
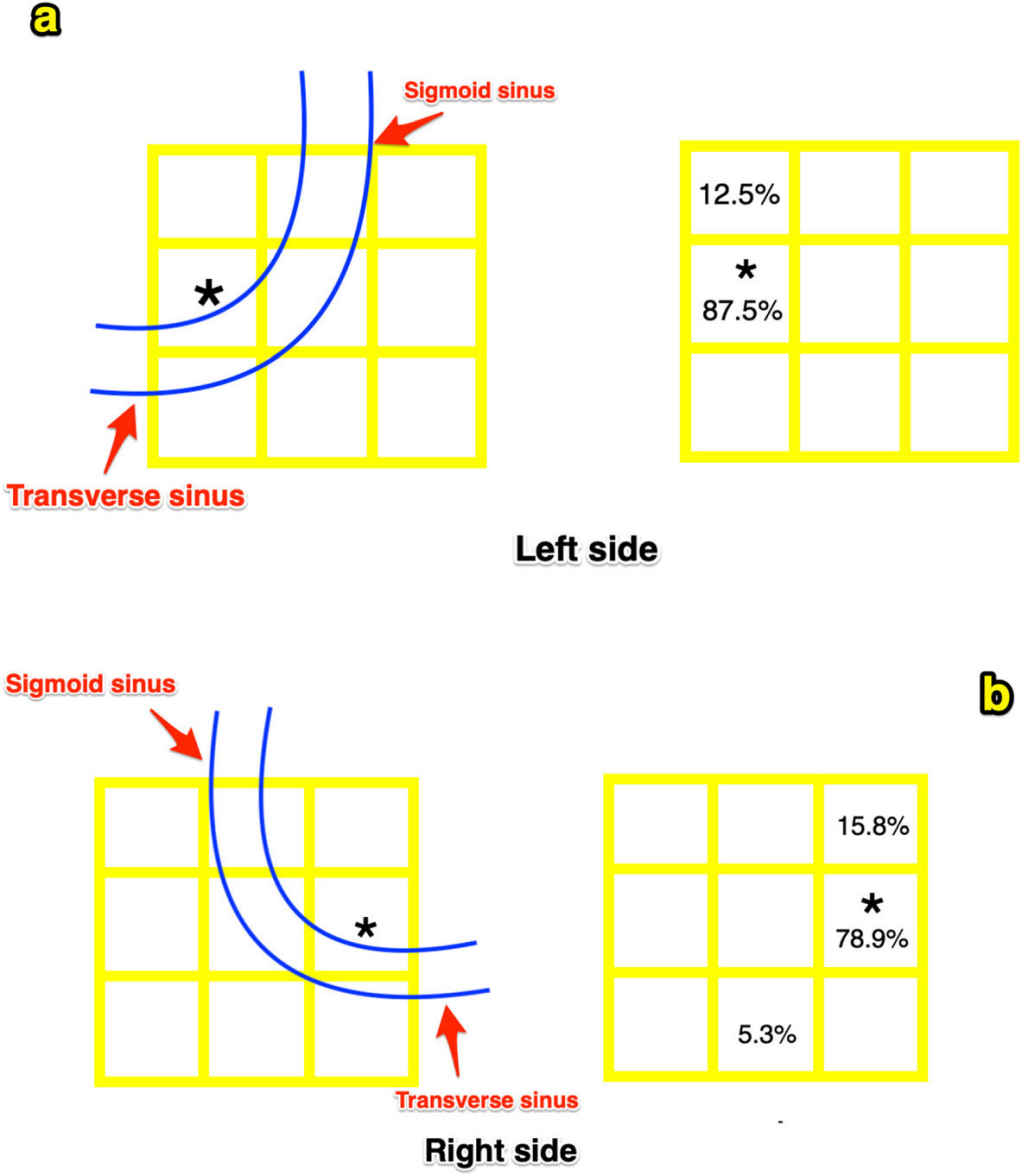
**a** The relationships between the projected TSSTs and the actual TSSTs on the left side are revealed. **b** The relationships between the projected TSSTs and the actual TSSTs on the right side are revealed. Each subdivision is 1 × 1 cm^2^. The asterisk symbol represents the projected TSST, and the locations of the actual TSSTs which were distributed into each subdivision were recorded as a percentage

The distance between the projected and the actual TSST exceeded 0.5 cm in 6 individuals (2 on the left and 4 on the right). These deviations are mostly shown along the axis from the pubic bone to the head and are rarely shown on the axis from the frontal bone to the occipital bone. The distributions of the actual TSST in the grid frame are revealed (Fig. 5). On the left, 2 projected TSSTs are inferior to the actual TSSTs by more than 0.5 cm. On the right, 3 projected TSSTs are inferior to the actual TSSTs by more than 0.5 cm, and 1 projected TSST is superior and posterior to the actual TSST by more than 0.5 cm.

## Discussion

RA has been widely used to perform surgeries regarding the cerebellopontine angle. For optimal visualization of the deep structures, a craniectomy should be made as close to the border of the transverse and the sigmoid sinus as possible. Currently, CT scan as a convenient and irreplaceable tool has been widely used to diagnose diseases. Given its special character which could vividly reveal bony structures, we proposed a technique that combines the existing anatomical knowledge regarding the posterior fossa and individualized high-resolution CT images to help identify the TSST.

To determine the location of the TSST, the asterion is a nonnegligible structure that can be defined as the intersection of the lambdoid suture, the occipitomastoid suture, and the parietomastoid suture. In the early periods, neurosurgeons always used the asterion as a landmark to perform the RA approach. However, a series of studies have shown that the asterion is unreliable due to its variability in location when it was used to guide RA craniectomies [9-11]. Because the lambdoid suture and the occipitomastoid suture are more visualizable than the asterion in high-resolution CT images, we thus shifted the focus of the investigation from the asterion to the skull suture.

The following section is about the location of the transverse sinus. Through cadaveric studies of the posterior fossa anatomical landmarks, Fukushima et al. [8] concluded that the LZI and the superior nuchal line (SNL) can indicate the inferior margin of the most distal part of the transverse sinus. By utilizing CT angiography images, Sheng et al. [7] suggested that the LZI and SNL are separate concepts and that the LZI is more reliable than the SNL regarding the location of the transverse sinus. In our study, 82.8% (29/35) of the LZIs indicated the inferior margin of the transverse sinus regardless of the side, which is consistent with the findings in previous literature; only 6 projected (2 on the left and 4 on the right, 6/35) transverse sinuses were superior or inferior by more than 0.5 cm from the actual transverse sinuses. Because the inion and the root of the zygoma can be clearly observed during a CT scan and the LZI can be easily marked during the operation, according to the results acquired from our study, we conclude that the LZI can be used to indicate the distal end of the transverse sinus.

The following section is about the location of the sigmoid sinus. After researching the relationships between the surface landmarks and the underlying structures, Ribas et al. [5] suggested that the posterior border of the sigmoid sinus can be determined by the intersection between the occipitomastoid suture and the imaginary line connecting the mastoid tip and the inion. Further, other authors [2, 3] who used high-tech and expensive devices such as the neural navigation and the 3-D volumetric image-rendered systems were able to obtain patient-specific anatomic information regarding the sigmoid sinus. Considering that sigmoid sinus can be embedded into a bone groove and anchored by the mastoid emissary vein, when these expensive devices are absent, a cost-effective and individualized technique should be proposed to control the risk of laceration of the sigmoid sinus. In our study, 97.1% (34/35) posterior borders of the sigmoid sinuses had been precisely determined, and only 1 projected sigmoid sinus was posterior to the actual sigmoid sinus on the right side. None of the sigmoid sinuses was lacerated during the craniectomies, and only 2 mastoid emissary veins, which were later coagulated efficiently, were stretched and started bleeding during the operations. Therefore, we recommend that our technique is performed with the assistance of high-resolution CT images that can obtain individualized anatomical information about the sigmoid sinus that is beneficial for RA craniectomies.

In our region, the mean expense of utilizing the neural navigation system is $726.78 (2018); for some special cases, the cost may be greater. However, the cost of high-resolution CT scanner is only $116.28 (2018), which is much more acceptable for some medical organizations than the former cost, and it only takes 20 min to obtain high-resolution CT images and analyze them preoperatively.

## Conclusion

Based on the results of our study, we conclude that our technique, with the assistance of high-resolution CT scans, is a cost-effective and reliable tool for identifying the location of the TSST which could be widely used to guarantee the safety of RA craniectomy.

## Data Availability

Not applicable

## Abbreviations

RA: Retrosigmoid approach
TSST: Transverse and sigmoid sinus transition
LZI: Line drawn from the root of the zygoma to the inion
CT: Computed tomography
FHP: Frankfurt horizontal plane
